# Developing strategies for reducing crashes and their social and health consequences in Qom ‎province 

**Published:** 2019-08

**Authors:** Babak Farzinnia, Hamidreza Khankeh, Bahram Mohaghegh, Hossain Khakie, Ali Khalaj

**Affiliations:** ^*a*^Qom University of Medical Sciences, Qom, Iran.; ^*b*^University of Social Welfare and Rehabilitation Sciences, Tehran, Iran.; ^*c*^Faculty of Health, Qom University of Medical Sciences, Qom, Iran.

## Abstract

**Background:**

Totally, 300 people died in traffic accidents in Qom province in 2018. Considering the special ‎geographical conditions and traffic culture, especially the vast use of motorcycles (40% of ‎deaths pertained to motorcyclists), the identification of strategies to reduce road accidents and ‎their consequences in Qom province is necessary.‎

**Methods:**

For the purposes of this study, a qualitative mode of research was carried out using action ‎research method in 2018. Semi-structured interviews were conducted with 22 participants who ‎were experts in road safety, emergency managing and social assistance. Then, content analysis ‎was used for categorization of explored solutions from expert opinions. The results were ‎presented at two meetings of provincial experts and university elites. And finally, strategies for ‎reducing road accidents and their consequences were categorized. ‎

**Results:**

The findings in the form of a final document was submitted to the Health Assembly of the ‎province for final approval and implementation. Results According to the opinions of experts in ‎Qom province, four strategies were identified as follows. 1- The strategy to improve the ‎behavior of the people of the province with an emphasis on increasing the perceived risk of road ‎accidents and empowering children, adolescents and adults. 2. The strategy for the development ‎of legal protections, including intensifying the treatment of high- risk drivers with violations ‎and empowering the law enforcement. 3. Integrated management and management strategy ‎including the use of the ability of insurers, charities and establish a permanent secretariat to ‎reduce road accidents and their consequences. 4. Urban and rural infrastructure reform ‎‎(transport, safety and relief) Including rehabilitation and development plans for the public ‎transport and clean-up fleet, intelligent monitoring and timely delivery (up-to-date), and ‎upgrading the quality of treatment after the scene of the crash. ‎

**Conclusions:**

The implementation of these strategies requires the participation of the public and the media. ‎Increasing the perceived risk of vehicle users and people, especially through documenting the ‎consequences of traffic accidents, such as disabilities, was identified as a key strategy. It is ‎recommended to assess the current situation before implementing any corrective interventions ‎and evaluate the effectiveness of the intervention after its implementation in the province.

**Keywords:**

Crashes, Reduction strategies, Action Research, Qom.‎

